# Short-term efficacy and safety of second generation bipolar transurethral vaporization of the prostate (B-TUVP) for large benign prostate enlargement: Results from a retrospective feasibility study

**DOI:** 10.1371/journal.pone.0261586

**Published:** 2021-12-16

**Authors:** Takeshi Fukazawa, Hiroki Ito, Masato Takanashi, Risa Shinoki, Tadashi Tabei, Takashi Kawahara, Francis X. Keeley, Marcus J. Drake, Kazuki Kobayashi

**Affiliations:** 1 Department of urology, Yokosuka Kyosai Hospital, Yokosuka, Japan; 2 Department of Urology, Yokohama City University Graduate School of Medicine, Yokohama, Japan; 3 Bristol Medical School and Bristol Urological Institute, Southmead Hospital, Bristol, United Kingdom; University Medical Center Utrecht, NETHERLANDS

## Abstract

**Background:**

To investigate the efficacy and safety of a second-generation bipolar transurethral electro vaporization of the prostate (B-TUVP) with the new oval-shaped electrode for large benign prostatic enlargement (BPE) with prostate volume (PV) ≥100ml.

**Materials and methods:**

100 patients who underwent second-generation B-TUVP with the oval-shaped electrode for male lower urinary tract symptom (LUTS) or urinary retention between July 2018 and July 2020 were enrolled in this study. The patients’ characteristics and treatment outcome were retrospectively compared between patients with PV <100ml and ≥100ml.

**Results:**

17/41 (41.5%) cases of PV ≥100ml and 24/59 cases (40.7%) of PV <100ml were catheterised due to urinary retention. The duration of post-operative catheter placement and hospital-stay of PV ≥100ml (3.1±1.3 and 5.6±2.3 days) were not different from PV <100ml (2.7±1.2 and 5.0±2.4 days). In uncatheterised patients (N = 59), post-void residual urine volume (PVR) significantly decreased after surgery in both groups, however, maximum uroflow rate (Q_max_) significantly increased after surgery only in PV <100ml but not in PV ≥100ml. Voiding symptoms and patients’ QoL derived from International Prostate Symptom Score (IPSS), IPSS-QoL (IPSS Quality of Life Index) and BPH Impact Index (BII) scores, significantly improved after B-TUVP in both groups. Catheter free status after final B-TUVP among patients with preoperative urinary retention was achieved in 18/24 (75.0%) and 14/17 (82.1%) cases in patient with <100ml and ≥100ml, respectively. There was no significant difference in post-operative Hb after B-TUVP, which was 97.0±5.4% of baseline for PV <100ml and 96.9±6.1% for PV ≥100ml and no TUR syndrome was observed.

**Conclusions:**

This is the first study investigating short-term efficacy and safety of second-generation B-TUVP with the oval-shaped electrode on large BPE. B-TUVP appears to be effective and safe for treating moderate-to-severe lower urinary tract symptoms and urinary retention in patients with large BPE.

## Introduction

The European Association of Urology (EAU) and the American Urological Association (AUA) guidelines indicate mono/bi-polar TURP as the standard option for the surgical treatment of moderate-to-severe lower urinary tract symptoms in men with prostate size of 30–80 ml [1, 2]. Bipolar transurethral vaporization of the prostate (B-TUVP) and laser vaporization of the prostate represent potential alternatives to TURP for men with PV of 30–80 ml, according to the EAU guidelines [2]. The AUA guidelines do not mention any restrictions on vaporization by prostate size [1].

Endoscopic management of large (PV > 80–100 ml) benign prostatic enlargement remains a clinical scenario with limited available treatment options (mainly enucleative), including, but not limited to, Holmium laser enucleation of the prostate (HoLEP) [1, 2]. RCT comparing HoLEP versus bipolar plasmakinetic enucleation of the prostate (BPEP) of large volume benign prostatic hyperplasia (BPH) (>80g) showed that HoLEP and BPEP are comparable regarding safety and efficacy for treatment of BPH, including for patients on anticoagulants [3]. However, BPEP required a longer catheterization duration and operative time [3]. Another RCT conducted to compare HoLEP versus bipolar plasmakinetic resection for large BPH (≥75g) showed that HoLEP had better safety profile, with significantly less operative duration, hemoglobin loss, hospital stay, and catheterization duration [4]. Although both procedures were effective, HoLEP showed significantly better percentage improvement of both IPSS and QoL [4]. To the best of our knowledge, there are no studies conducted to date evaluating efficacy and safety of B-TUVP vs other endourological modalities in men with prostates larger than 80 mL. Hence, the position of B-TUVP for the treatment of large BPH still remains unclear.

**Fig 1:**
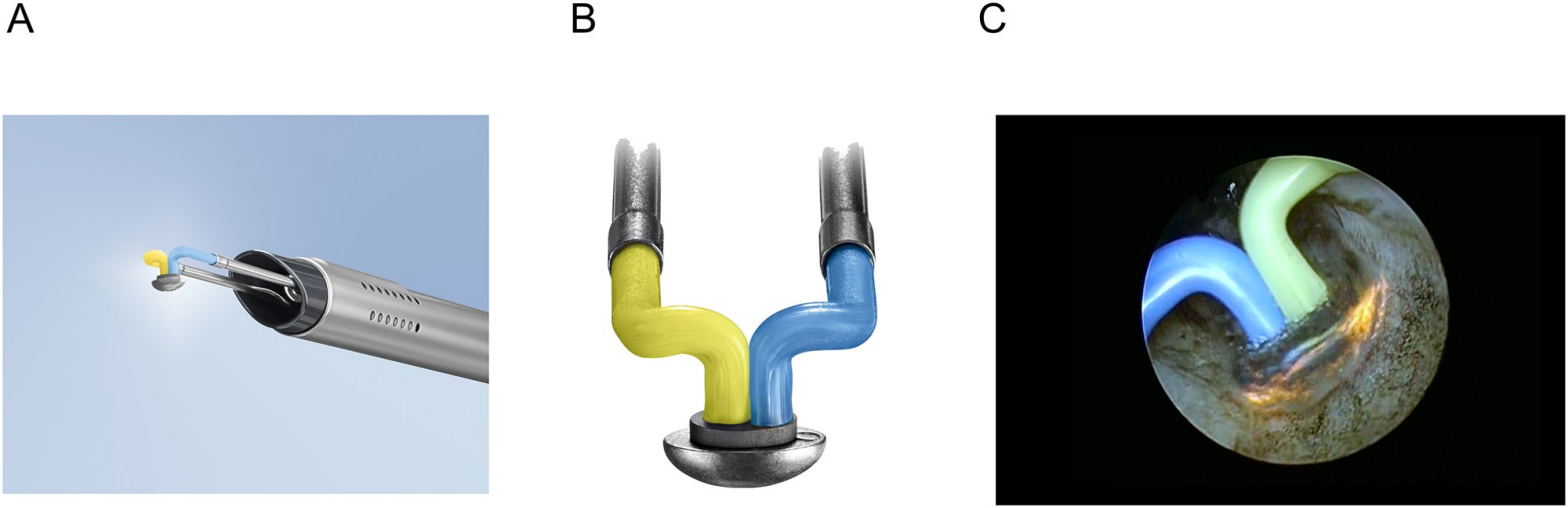
Image of the second-generation B-TUVP with oval electrode (A, Olympus, Tokyo) and zoomed image of the oval electrode (B) (Both images were kindly provided with request for citation by Olympus, Tokyo).Image of vaporization of prostate with the oval electrode in second-generation B-TUVP (C).

In 2018, the new oval-shaped electrode (Olympus, Japan) was developed (Fig 1A and 1B, Olympus, Japan) as a second-generation B-TUVP electrode, with a large width (4.0mm) enabling a wide vapor pocket around the electrode, and thus improved efficacy of vaporization of prostate tissue (Fig 1C). We hypothesized this novel oval electrode may allow for more effective treatment of large prostatic enlargement (BPE) and evaluated the efficacy and safety of this potentially improved electrode for large BPE of more than 100ml in this study.

## Materials and methods

### Study population

106 consecutive patients underwent second-generation B-TUVP with the oval-shaped electrode (Fig 1A and 1B) between July 2018 and July 2020 at Yokosuka Kyosai Hospital. B-TUVP was performed by a total of 6 senior urologists. Patients with suspected impaired detrusor function, active urinary tract infection, prostate cancer, bladder cancer, urethral stricture or dementia who were not able to complete outcome measurement were excluded. Thereby, six patients (2 prostate cancer, 3 dementia and 1 in terminal stages of non-prostate cancer) were excluded from this study. The remaining 100 patients were retrospectively enrolled. PV were measured by abdominal ultrasound scan or prostate MRI scan. The study was conducted in accordance with the principles set out in the Declaration of Helsinki and all local regulations. The study protocol (IRB number YKH20-74) waiving the requirement for written informed consent was approved by the institutional ethics committee of Yokosuka Kyosai Hospital. Informed consent was obtained in the form of an opt-out on the Yokosuka Kyosai Hospital web site. The new oval-shaped electrode (Olympus, Japan) was certificated for clinical use (certification number: 29ABBZX00048000, 26^th^ Jun. 2017) after clinical safety tests conducted by TŰV Rheinland Japan Ltd. (Yokohama, Japan), the official third company entrusted by the Japanese Ministry of Health, Labor and Welfare. No previous feasibility and safety (phase 1) studies have been carried out with this device.

Surgical indications were based on the following; the presence of moderate to severe lower urinary tract symptoms (LUTS) (International Prostate Symptoms Score (IPSS) >7) despite proper medication, maximum flow rate (Q_max_) <10 mL/s, persistent or recurrent urinary retention or bladder stones, or post-void residual urine volume (PVR) >100 ml. The patients with prostate-specific antigen (PSA) ≥10 ng/ml were recommended to undergo prostate MRI and if that showed suspected prostate cancer, they were recommended to undergo needle biopsy before B-TUVP. The patients with PSA 4–10 ng/ml were recommended to undergo prostate MRI based on patient preference. If that showed suspected prostate cancer, they were recommended to undergo needle biopsy before B-TUVP. For patients on anticoagulant/antiplatelet therapy, we consulted the prescribing physicians and anaesthetists before B-TUVP. Anticoagulant/antiplatelet therapy were stopped for B-TUVP if the physicians and anaesthetists decided the patient’s status allowed this, and medications were resumed if hematuria was absent. When they recommended continuing anticoagulant/antiplatelet therapy, we informed the patient thoroughly of possible higher risk of perioperative bleeding before B-TUVP. When the patient wanted to undertake B-TUVP despite of higher risk of bleeding, we performed B-TUVP as usual surgical procedure under anticoagulant/antiplatelet therapy. We elected not to perform cystometry and pressure flow studies for patients in this study.

The patients’ characteristics and treatment outcomes were retrospectively compared between patients with PV <100ml and ≥100ml. Haemoglobin (Hb) was measured at pre-operative baseline and at 1 POD (post-operative day). Main treatment outcomes were measured by IPSS, IPSS Quality of Life Index (IPSS-QoL), overactive bladder symptom score (OABSS) [5], the Benign Prostatic Hyperplasia Impact Index (BII) [6], uroflowmetry tests (voided volume and Q_max_) and PVR (at pre-operative baseline and 1, 3 and 6 POM (post-operative months)). Serum PSA value was also evaluated at pre-operative baseline and 3 POM.

The OABSS, originally developed in Japan, is a 4-item questionnaire that expresses OAB symptoms on a single scale [5]. The OABSS question items address the following individual symptoms: daytime frequency, nocturia, urgency, and urgency incontinence. Gotoh et al. reported that the OABSS was useful for assessing the effects of treatment on OAB symptoms and was responsive to treatment-related changes [7]. The BII, developed by the American Urological Association, assesses the impact of BPH symptoms on patient health and functioning [6]. The BII is a self-administered questionnaire with 4 questions about urinary problems during the past month regarding physical discomfort, worry about health, how bothersome symptoms are, and whether the symptoms are interfering with doing usual activities.

### Operative procedure

**Fig 2:**
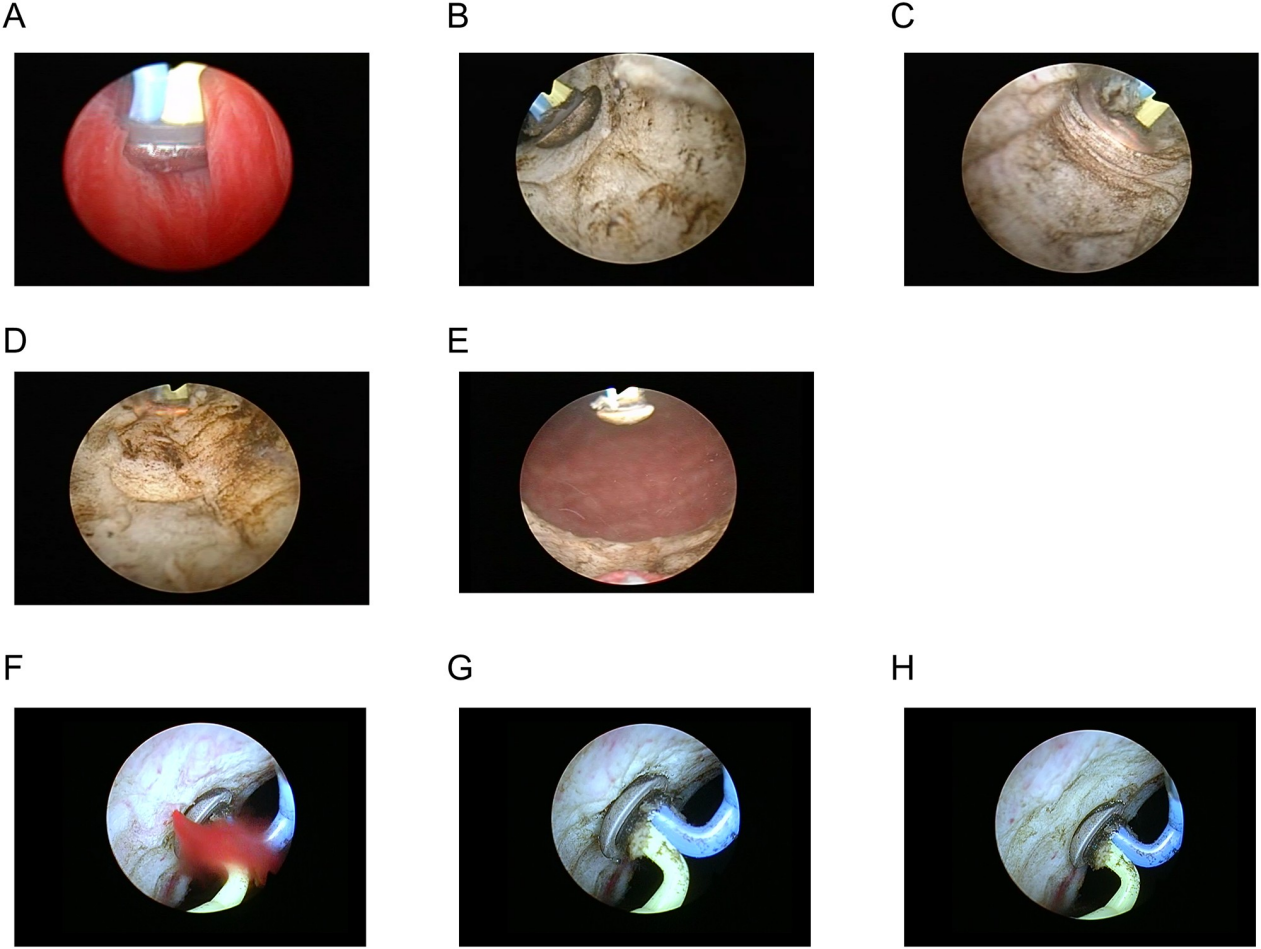
After careful observation of the enlarged prostate (A), the vaporization procedure started with the creation of a working space at 5 (B) and 7 o’clock (C). Following vaporization of the middle (D), and lateral lobes, well-opened prostate and bladder neck were confirmed (E). The coagulation setting could rapidly be deployed with the same electrode, and even arterial bleeding (F) could be terminated immediately (G, coagulation with oval-shaped electrode, H, confirmation of cessation of bleeding).

A preoperative antibiotic, a 3^rd^ generation cephem, was administered before surgery. Routine urine culture was not performed and specific antibiotics were used according to the urine culture for patients with a history of urinary tract infection before B-TUVP. After spinal or general anesthesia was applied, the patient was placed in the lithotomy position and sterile draped. A 26 Fr continuous flow resectoscope (30° cystoscopic lens) was inserted into the urethra under direct vision to observe the urethral status, prostatic enlargement and bladder neck status. An oval-shaped electrode (Olympus, Japan) was used for vaporization of the prostate. Normal saline was irrigated with a 26 Fr resectoscope, with the TURis system (Olympus), using cutting/coagulation settings of 200W/120W [8]. After identifying the ureteral orifices, the verumontanum and external urethral sphincter, the outline of the enlarged prostate was carefully observed (Fig 2A). The vaporization procedure started with the creation of a working space at 5 (Fig 2B) and 7 o’clock (Fig 2C). Then, the middle (Fig 2D) and lateral lobe were vaporized. Vaporization was carried out with the cutting setting of 200W. After finishing vaporization, complete opening of the prostate and bladder neck were confirmed (Fig 2E). Then an 18Fr 3-way transurethral catheter was placed, and this catheter was removed when hematuria had resolved (approximately 2–4 POD).

To minimize bleeding during the procedures, the coagulation setting could rapidly be deployed with the same electrode, and even arterial bleeding could be terminated immediately (Fig 2F–2H). In case of an enlarged prostatic middle lobe with protrusion into the bladder, changing the electrode to a loop-shaped electrode enabled more efficient resection.

### Statistical analysis

Statistical analysis was undertaken using SPSS software (SPSS version 22, Inc., Chicago, IL). Paired and un-paired student’s t test and Dunnet’s test were used, as appropriate, to compare preoperative and postoperative continuous variables between the groups. The chi-square and Fisher’s exact tests were used as appropriate to compare discrete variables. Continuous variables are presented as mean and standard deviation, and discrete variables are presented as percentages. P values <0.05 were considered significant.

## Results

Table 1 shows the patients’ backgrounds. Among the 100 cases, 41 patients had PV more than 100ml (average PV (±SD) was 137.6±33.4 ml) and 59 cases had PV less than 100ml (average PV (±SD) was 75.1±49.3 ml). Twenty four and seventeen patients were catheterised due to urinary retention in patients with prostates <100ml and ≥100ml, respectively; there was no significant difference in urinary retention rate between the 2 groups (40.7% and 41.5%). Heart disease was significantly more prevalent in patients with PV ≥100ml (13 ischemic heart disease and 3 atrial fibrillation) than those with PV <100ml (5 ischemic heart disease and 2 atrial fibrillation, P<0.001). Eighteen patients had anticoagulant/antiplatelet therapy and 17 patients stopped taking it before surgery. Only 1 case with PV <100ml underwent B-TUVP while still on anticoagulants due to high risk of recurrent cerebral cortex infarction; this did not result in any bleeding event after B-TUVP.

**Table 1:** Patients’ backgrounds.

All patients (N = 100)	PV<100ml	PV≥100ml	p-value *
Number of patients	59	41	
Age (years-old)	75.8±7.4	74.8±6.9	0.481
Prostate volume (ml)	75.1±49.3	137.6±33.4	<0.001
PSA (ng/mL)	6.8±7.6	14.1±14.9	<0.001
Urinary retention	24	17	0.748
Past medical history			
Hypertension	25	17	0.928
Cerebrovascular events	8	3	0.326
Diabetes mellitus	9	10	0.252
Heart disease	7	16	<0.001
Chronic obstructive pulmonary disease	5	5	0.541
Herniated intervertebral disc	7	8	0.292
Medications prior to B-TUVP (N = 100)			
Alpha-1 blockers	45	26	0.163
5-α reductase inhibitor	15	13	0.491
Anticoagulant/antiplatelet therapy	10	8	0.108

* Compared patients with prostates <100ml versus ≥100ml.

All continuous values are presented as mean±SD.

PV: prostate volume.

PSA: prostate-specific antigen.

Surgical outcomes of B-TUVP, uroflowmetric findings and patient reported outcomes are summarized in Tables 2 and 3. Total endoscopic time of the PV≥100ml group was significantly longer than for PV<100ml. Catheter free status after first B-TUVP for patients with preoperative urinary retention was achieved in 18/24 (75.0%) and 10/17 (58.8%) cases in patient with <100ml and ≥100ml, respectively. Four patients with PV≥100ml received a second B-TUVP because of persisting urinary retention and all 4 cases then obtained catheter free status. Hence, catheter free status after final B-TUVP was 14/17 (82.1%) in patients with ≥100ml (Table 2).

**Table 2:** Surgical outcome of B-TUVP.

		PV<100ml	PV≥100ml	p-value*
		(N = 59)	(N = 41)	
Operative time (total endoscopic time, mins)		99.2±25.1	123.7±25.1	<0.001
Hemoglobin (g/dL)	pre	13.6±1.4	13.6±1.9	0.941
	1POD	13.1±1.3	13.1±1.7	0.997
	Change rate (%)	97.0±5.4	96.9±6.1	0.989
Duration of post-operative catheter implantation (days)		2.7±1.2	3.1±1.3	0.093
Hospital stay period after B-TUVP (days)		5.0±2.4	5.6±2.3	0.296
PSA	pre	6.8±7.6	14.1±14.9	0.003
	3POM	4.0±4.9	7.1±6.5	0.028
	Change rate (%)	53.9±21.4	58.6±36.9	0.525
Necessity of 2nd procedure		0	4 (10%)	0.014
Achieving catheter free status among patients with preoperative urinary retention	After first B-TUVP	18/24 (75.0%)	10/17(58.8%)	0.118
	After final B-TUVP	Same as above	14/17(82.4%)	0.575

* Compared between patients with prostates <100ml and ≥100ml.

Values were presented as mean±SD or Number of cases (%).

POD: post-operative day.

POM: post-operative months.

PSA: prostate-specific antigen.

**Table 3:** Uroflowmetric parameters and patient report outcomes before and after B-TUVP of uncatheterised patients (N = 59).

Uncatheterised patients (N = 59)		PV<100ml	p-value *	PV≥100ml	p-value *
		(N = 35)		(N = 24)	
Q_max_ (ml/sec)	pre	8.9±5.9		10.2±6.0	
	1POM	11.9±6.0	0.245	13.1±5.9	0.420
	3POM	13.5±8.1	0.040	11.8±6.4	0.718
	6POM	14.3±10.6	0.020	13.2±7.2	0.321
Q_ave_ (ml/sec)	pre	4.8±2.4		5.2±2.6	
	1POM	6.9±3.1	0.022	7.1±2.6	0.165
	3POM	7.4±3.4	0.004	6.6±2.6	0.238
	6POM	7.0±3.7	0.024	7.3±3.9	0.067
Voided volume (ml)	pre	118.9±100.9		167.2±110.3	
	1POM	117.9±80.5	0.990	151.4±82.4	0.953
	3POM	155.7±144.1	0.433	144.8±108.0	0.817
	6POM	154.1±128.4	0.516	185.6±124.4	0.914
PVR (ml)	pre	173.9±230.1		208.4±258.2	
	1POM	82.8±91.9	0.030	59.8±46.0	0.012
	3POM	70.2±98.5	0.012	93.0±66.0	0.021
	6POM	53.8±67.1	0.005	87.4±56.8	0.039
Total IPSS	pre	22.0±7.8		17.5±7.6	
	1POM	13.9±8.5	0.002	12.9±7.3	0.063
	3POM	11.1±10.0	<0.001	8.6±5.2	<0.001
	6POM	7.8±8.8	<0.001	6.2±3.9	<0.001
IPSS-QoL	pre	4.9±1.1		4.7±1.0	
	1POM	3.8±1.9	0.030	3.5±1.5	0.022
	3POM	2.6±2.0	<0.001	2.8±1.4	<0.001
	6POM	2.0±1.7	<0.001	2.5±2.0	<0.001
BII	pre	6.2±3.7		6.4±3.5	
	1POM	4.6±3.8	0.298	3.6±3.4	0.025
	3POM	3.5±4.2	0.026	2.3±2.8	0.001
	6POM	2.2±3.7	0.001	2.3±3.6	0.001
OABSS	pre	6.9±3.1		6.5±3.0	
	1POM	7.1±4.1	0.987	7.5±3.6	0.741
	3POM	5.4±3.8	0.301	5.6±3.3	0.780
	6POM	3.9±3.1	0.012	3.7±2.1	0.069

* Compared to preoperative baseline value in each group with prostates <100ml and ≥100ml.

All continuous values are presented as mean ± SD.

PV: prostate volumes.

POM: post-operative months.

Q_max_: Maximum uroflow rate.

Q_ave_: Average uroflow rate.

PVR: post-void residual urine volume.

IPSS: International Prostate Symptom Score.

IPSS-QoL: IPSS Quality of Life Index.

BII: Benign Prostatic Hyperplasia Impact Index.

OABSS: Overactive bladder symptom score.

For uncatheterized patients, maximum uroflow rate (Q_max_) significantly increased after surgery in the PV <100ml group but not in the PV≥100ml group at 6POM (Table 3). PVR, total IPSS and IPSS-QoL were significantly lower after surgery in both ≥100ml and <100ml groups (Table 3).

In catheterized patients who achieved catheter free status after B-TUVP, there was no significant difference in Q_max_ at 6POM between patients from the 2 groups (16.2 ml/s in PV≥100ml and 12.4 ml/s in PV<100ml, P = 0.266), but there was significant difference in PVR at 6POM (134.8ml in PV≥100ml and 61.8ml in PV<100ml, P = 0.038). Total IPSS (2.0±1.8) and IPSS-QoL (0.9±0.9) of the PV≥100ml patients were significantly lower than total IPSS (6.3±4.0, P = 0.007) and IPSS-QoL (2.3±0.9, P = 0.002) for the smaller prostate volumes.

In terms of post-operative complications, post-operative fever arose in 7 cases of PV ≥100ml and 14 cases of <100ml (N.S.) (Table 4). There were 2 cases of septic shock (Clavien-Dindo Grade IVb) needing catecholamine therapy. Post-operative bleeding (Clavien-Dindo Grade I) (prolonged gross hematuria more than 3 days after B-TUVP) occurred in 7 cases from the PV ≥100ml group and 14 cases from the smaller prostate group (N.S.) (Table 4). One case from the PV ≥100ml group needed to return to theatre for transurethral coagulation, while the rest of them were treated conservatively (Clavien-Dindo Grade IIIb). No blood transfusion was needed and no TUR syndrome occurred. 4/100 procedures required a change of electrode from oval-shaped to looped-shape during B-TUVP for treating an enlarged middle lobe protruding into the bladder. All 4 procedures were performed without any surgical complications. In all procedures, a single oval-shaped electrode was used per surgery. No clinical indicator of de novo urethral stricture was identified at 6POM.

**Table 4:** Post-operative complications following B-TUVP.

	PV<100ml	PV≥100ml	p-value
	(N = 59)	(N = 41)	
Post-operative fever	14(24%)	7(17%)	0.457
Continuous post-operative hematuria	14(24%)	7(17%)	0.457
Post-operative sepsis	0	2(5%)	0.087
Post-operative cerebral cortex infarction	0	1(2%)	0.228

All values were presented as Number of cases (%).

## Discussion

This study was a first retrospective feasibility study showing the high efficacy and reliable safety of a second-generation B-TUVP for treating substantial prostatic enlargement, as well as mild-moderate enlargement. In uncatheterized patients, PVR significantly decreased after surgery in both PV ≥100ml and <100ml groups, however, Q_max_ significantly increased after surgery only where PV was <100ml. Voiding symptoms and patients’ QoL derived from IPSS, IPSS-QoL and BII, clearly and significantly improved after B-TUVP in both groups. The hospital stay duration and postoperative catheter duration for did not differ significantly between the groups, indicating the clinical feasibility of B-TUVP for large BPE. Furthermore, second-generation B-TUVP utilizes widely available surgical systems, i.e. the bipolar TUR system [8–13], which is already used widely for the treatment of large BPE [14] and bladder tumors [15], bringing potential cost saving [16, 17] and easy equipment availability.

The evidence for managing patients with urinary retention associated with benign prostatic obstruction using pharmacological or nonpharmacological treatments is limited [18]. Some RCTs showed alpha1-blockers provided significantly higher rates of successful trial without catheter compared with placebo for the treatment of mild-moderate BPH [18]. Compared to those findings, B-TUVP achieved promising surgical outcomes for catheterized patients, notably no further need of medication after surgery, potentially delivering QoL improvement.

This study revealed a possible drawback of B-TUVP for treatment of PV ≥100ml compared to PV <100ml. Two sepsis cases were confirmed in PV ≥100ml and none in PV <100ml, indicating potentially a greater risk, possibly because of longer operative duration. Use of a second procedure was only done in the PV ≥100ml group. All 4 such cases achieved catheter free status after 2^nd^ B-TUVP, suggesting that staged B-TUVP might be a clinical treatment strategy for PV ≥100ml.

The EAU and AUA recommend laser treatment (enucleation or vaporization) for patients on anticoagulant/antiplatelet therapy [1, 2]. The EAU recommends laser modalities: Tm:YAG laser enucleation and laser vaporization of the prostate using 80-watt 532-nm Potassium-Titanyl-Phosphate (KTP) or 120- or 180-watt 532-nm Lithium Borat (LBO) lasers [2]. Except for one case, we stopped anticoagulant/antiplatelet before B-TUVP as far as was safely possible. Hence, this study did not investigate the safety of B-TUVP whilst using anticoagulant/antiplatelet therapy. A further study would be needed to address this point.

This study revealed higher prevalence of heart disease in patients with PV ≥100ml than those with PV <100ml. Some previous studies showed that the occurrence of coronary artery disease and atrial fibrillation is significantly higher among patients with BPH [19–21]. Speculatively, it has been suggested that smooth muscle proliferation, insulin, inflammation and metabolic syndrome may play central roles in pathogenesis of both BPH and heart disease [22].

Limitations of the current study include its retrospective nature and the short duration of follow-up. The small number of enrolled patients may mean it was underpowered for some parameters in statistical analysis. A randomised study with longer follow-up will be needed to confirm the long-term efficacy of B-TUVP. In this study, only a few cases were followed more than 6 months, though S1 Fig shows widely opened prostate and bladder neck even 18 months after B-TUVP.

## Conclusions

This is first retrospective feasibility study investigating efficacy and safety of second-generation B-TUVP using an oval-shaped electrode for large BPE of more than 100ml. B-TUVP is clinically effective for the relief of LUTS and urinary retention of patients with large BPE without severe adverse events.

## Supporting information

S1 Fig (A) Flexible cystoscopic appearance of the vaporized prostate and well opened bladder neck at 18 months post-operatively for a patient who underwent B-TUVP. (B) Retrograde view during flexible cystoscopy.
(TIF)

S1 Data (XLS)

## Abbreviations

AUA: American Urological Association
BII: BPH Impact Index
BPE: benign prostatic enlargement
BPEP: bipolar plasmakinetic enucleation of the prostate
BPH: benign prostatic hyperplasia
B-TUVP: bipolar transurethral electro vaporization of the prostate
EAU: European Association of Urology
Hb: Hemoglobin
HoLEP: Holmium laser enucleation of the prostate
IPSS: International Prostate Symptom Score
IPSS-QoL: IPSS Quality of Life Index
LUTS: Lower urinary tract symptoms
N.S.: not significant
OABSS: Overactive Bladder Symptom Score
POD: post-operative day
POM: post-operative months
PROs: patient reported outcomes
PSA: prostatic-specific antigen
PV: prostate volumes
PVP: photoselective vaporisation of the prostate
PVR: post-void residual urine volume
Qave: Average uroflow rate
Qmax: Maximum uroflow rate
SP: simple prostatectomy
TURP: transurethral resection of the prostate
