# Supplementary figures and images for: Short-term efficacy and safety of second generation bipolar transurethral vaporization of the prostate (B-TUVP) for large benign prostate enlargement: Results from a retrospective feasibility study
Source: PLoS One. 2021 Dec 16;16(12):e0261586. doi: 10.1371/journal.pone.0261586 (PMC8675650; doi:10.1371/journal.pone.0261586)

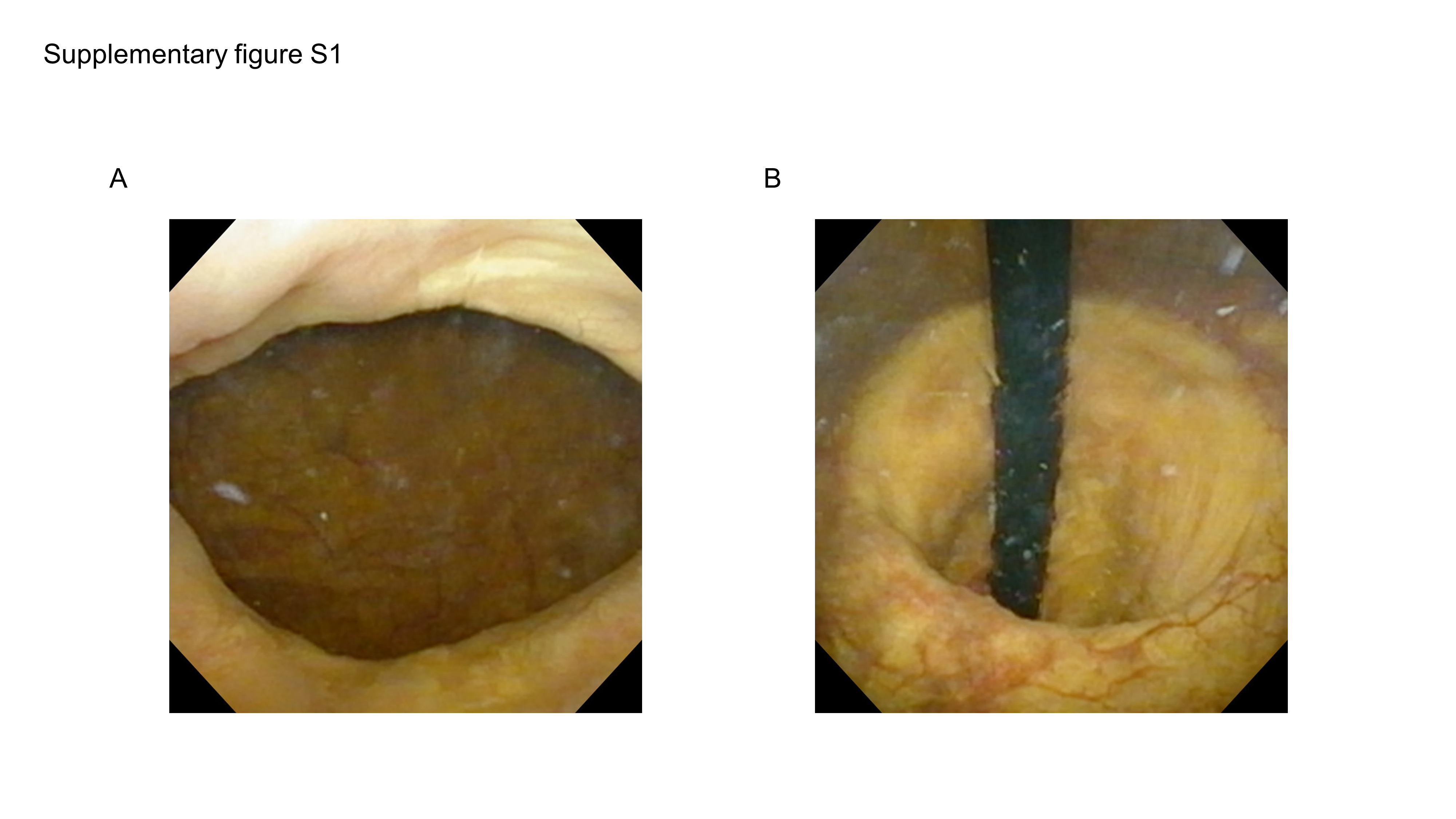

Supplement: S1 Fig — (A) Flexible cystoscopic appearance of the vaporized prostate and well opened bladder neck at 18 months post-operatively for a patient who underwent B-TUVP. (B) Retrograde view during flexible cystoscopy. (TIF) [file pone.0261586.s001.TIF]
